# QTL analysis uncovers the genetic architecture of resistance to chocolate spot disease caused by four *Botrytis* species on faba bean

**DOI:** 10.1186/s12870-026-08699-0

**Published:** 2026-04-10

**Authors:** Maniruzzaman, Marja Jalli, Alan H. Schulman, Hamid Khazaei, Frederick L. Stoddard

**Affiliations:** 1 Department of Agricultural Sciences, University of Helsinki, (Latokartanonkaari 5-7) FI-00014, PO Box 27, Helsinki, Finland; 2 Natural Resources Institute Finland, Latokartanonkaari 9, 00790, Helsinki, Finland; 3 Institute of Biotechnology and Viikki Plant Science Centre, University of Helsinki, Helsinki, Finland

**Keywords:** Gene mapping, Quantitative trait loci, Candidate gene, Disease resistance, *Botrytis* spp.

## Abstract

**Background:**

Chocolate spot (CS), caused by *Botrytis* species, is a major disease constraining faba bean production worldwide. We investigated the genetic basis of resistance to four *Botrytis* species (*B. fabae, B. cinerea*, *B. pseudocinerea*, and *B. fabiopsis*) using a recombinant inbred line (RIL) population derived from the cross Mélodie/2 × ILB 938/2, which was evaluated with a detached-leaf assay.

**Results:**

Significant variation in disease severity was observed among parental lines and RILs for all four *Botrytis* species. The detached-leaf assay showed a strong correlation with a whole-plant assay conducted using a different *B. fabae* isolate, validating its effectiveness as a screening tool. QTL analysis identified six loci associated with response to *Botrytis* infection across Chr1, Chr4, and Chr6, explaining 7–11% of the total phenotypic variance. The overlapping QTLs on Chr1 conferred partial resistance to all four *Botrytis* species, while QTLs on Chr4 and Chr6 were specific to *B. fabae*. None of the other three species showed a unique QTL. Putative candidate gene analysis within QTL intervals revealed several defense-related gene families, including F-box protein, WRKY, MYB, and AP2/ERF transcription factors, and peroxidases involved in oxidative stress regulation.

**Conclusion:**

The consistency of response across species and the lack of unique responses from the non-*fabae* species suggests that selection for resistance to *B. fabae* may also select resistance to other *Botrytis* species. This study provides new insights into the shared and species-specific genetic architecture of *Botrytis* resistance in faba bean.

**Supplementary Information:**

The online version contains supplementary material available at 10.1186/s12870-026-08699-0.

## Introduction

Faba bean (*Vicia faba* L.) is an ancient grain legume crop with a large genome (2n = 2x = 12, ∼13 Gb), predominantly cultivated as a source of protein for both human and animal consumption in temperate and subtropical regions [1, 2]. Its seeds are rich in protein, dietary fibre, and micronutrients [3]. Faba bean cultivation enhances soil fertility through its substantial ability of biological nitrogen fixation, thereby reducing the need for synthetic fertilizers in crop production and contributing to more sustainable agricultural systems [4]. The cultivation of faba bean has been constrained by its susceptibility to a range of abiotic and biotic stresses [5, 6]. Among the biotic constraints, chocolate spot (CS) is a major and widespread disease, capable of causing complete yield loss in susceptible cultivars under favorable conditions [7, 8].

CS disease is caused primarily by *Botrytis fabae* (Sard.). Three other *Botrytis* species, namely *B. cinerea*, *B. pseudocinerea,* and *B. fabiopsis,* are now known to cause the disease [9]. *B. fabae* has a narrow host range [10], whereas *B. cinerea* infects a broad range of host plants. Another distinguishing characteristic is that *B. fabae* is typically found on leaves, stems, and pods, while *B. cinerea* is primarily associated with floral parts [11]. *B. fabiopsis* produces symptoms similar to *B. fabae* [8] and can coexist with both *B. fabae* and *B. cinerea*, potentially infecting hosts in a complex manner. *B. cinerea* and *B. pseudocinerea* are morphologically identical but genetically dissimilar [12], and *B. pseudocinerea* produces larger lesions on detached faba bean leaves [13, 14].

CS disease can be managed through the application of protective or preventive fungicides. While chemical control can be effective in the short term, it is costly, not environmentally friendly, labor-intensive, and may lead to the development of fungicide-resistant pathogen strains. Genetic improvement of faba bean cultivars for resistance offers a more sustainable and long-term solution [15]. Breeding for CS resistance has been challenging because it is a complex trait [16] controlled by many genes with minor effects [17, 18]. So far, only partial resistance to CS has been identified in faba bean germplasm [19]. Development of DNA markers can accelerate genomics-assisted breeding for CS resistance in this crop.

The faba bean genome has been sequenced and assembled [1], and a pan-genome is in preparation [20, 21]. Next-generation sequencing technologies have allowed large-scale mining of DNA markers such as single-nucleotide polymorphisms (SNPs) for this crop [22–24]. Recently developed rich genomic and genetic resources need to be combined with novel phenotyping to identify QTLs, candidate genes and pathways associated with the desired traits. These efforts enable understanding of the biological functions, mining of the responsible alleles, and development of molecular markers that will enable breeders to accelerate cultivar development for environmental stressors such as CS. The first attempt at gene mapping for CS in faba bean was conducted by Gela et al. [17] using an advanced bi-parental population derived from the cross Mélodie/2 × ILB 938/2. A seven-way cross was also used for this purpose by Skovbjerg et al. [25]. In both studies, ILB 938 was used as a source of partial resistance to CS, and high-density Axiom SNP genotyping arrays were employed for genotyping [22]. Webb et al. [18] employed a multi-generation mapping population developed from the cross Maris Bead (partial resistance to CS) × IG 70726 using DArT genotyping. These studies identified several minor-effect genomic regions across all faba bean chromosomes, with loci on chromosomes 1 and 6 (Chr1 and Chr6) being consistent across reports. All of these studies used *B. fabae* as the main *Botrytis* species responsible for causing CS in faba bean [11]. To date, there have been no reports on the QTL mapping of the other *Botrytis* species in faba bean. Thus, the main aim of this study was to investigate the genetic basis of resistance to *B. fabae*, *B. cinerea*, *B. pseudocinerea,* and *B. fabiopsis* in an extensively genotyped faba bean mapping population. By using detached leaves with four leaflets, we synchronized both the inoculation and the physiological age of the inoculated part, removing some potential sources of background variability.

## Materials and methods

### Plant material

A set of 165 recombinant inbred lines (RILs) from the Mélodie/2 × ILB 938/2 mapping population [26] at the F_8_ generation was used for this study. Mélodie/2 is a low-vicine-convicine French cultivar with high efficiency in water use but is susceptible to CS. ILB 938/2 (IG 12132) is a landrace originating from the Andean region of Colombia and Ecuador, and carries resistance or tolerance to several stresses and diseases, including CS [27]. The Finnish cultivar, Kontu, was included in all experiments as a susceptible check [13].

### Growing conditions

Five seeds from each RIL were grown in a 2.5 L pot filled with peat soil (Kekkilä Coarse potting mix WR8494, Kekkilä-BVB OY, Vantaa, Finland). Seeds were inoculated before sowing with *Rhizobium leguminosarum* biovar. *viciae* (Elomestari Oy, Tornio, Finland). Plants were maintained and grown for 6–8 weeks in a controlled-environment growth chamber (Weiss-2000, Weiss Technik GmbH, Reiskirchen-Lindsruth, Germany). The temperature was set to 22 °C during the day and 20 °C at night, with a 12-h photoperiod under visible light (150 μmol m⁻^2^ s⁻^1^ photosynthetic photon flux density, PPFD).

### Development of species

The *Botrytis* isolates were collected from the Institute of Soil and Plant Sciences, Latvia University of Life Sciences and Technologies, Latvia, and were sequenced and identified using three nuclear genes, namely, RNA polymerase II (RPB2), Heat shock protein 60 (HSP60) and Glyceraldehyde 3-phosphate dehydrogenase (G3PDH) [14]. The virulence of these *Botrytis* species was confirmed by Maniruzzaman et al. [13], and the most virulent of each, namely *B. fabae* (19B053-4)*, B. cinerea* (19B048)*, B. pseudocinerea* (18B11) and *B. fabiopsis* (19B175) [13, 14], was chosen for this study. They were cultured on half-strength potato dextrose agar (PDA) and incubated in darkness at room temperature (20 °C) for 7 days, then placed for 12 h under near-ultraviolet light (NUV) and 12 h in the dark for 7 days to induce sporulation. The cultured plates were flooded with sterile distilled water and scraped with a triangular steel rod to dislodge the spores. The suspension was filtered through two layers of cheesecloth into a 250 ml conical flask. Tween 20 was then added to a final concentration of 0.03% (v/v) and the suspension was gently mixed to obtain a homogeneous spore suspension. Spore concentration was determined using a Fuchs–Rosenthal hemocytometer (Assistent, Germany) under a light microscope (Zeiss Imager.M2, Oberkochen, Germany). The number of spores was counted in ten large squares of the hemocytometer (each 0.1 µl), then multiply it by the correction factor (10/11), and the average count per square was used to calculate the initial spore concentration (C_1_, spores/ml) using standard hemocytometer procedures. The suspension was diluted to the desired final concentration of 4 × 10^5^ spores ml⁻^1^ (C_2_) using sterile distilled water, applying the dilution equation *C*_*1*_*V*_*1*_ = *C*_*2*_*V*_*2*_. All calculations were performed explicitly for each preparation to ensure accurate and reproducible spore concentration.

### Disease screening

**Fig. 1:**
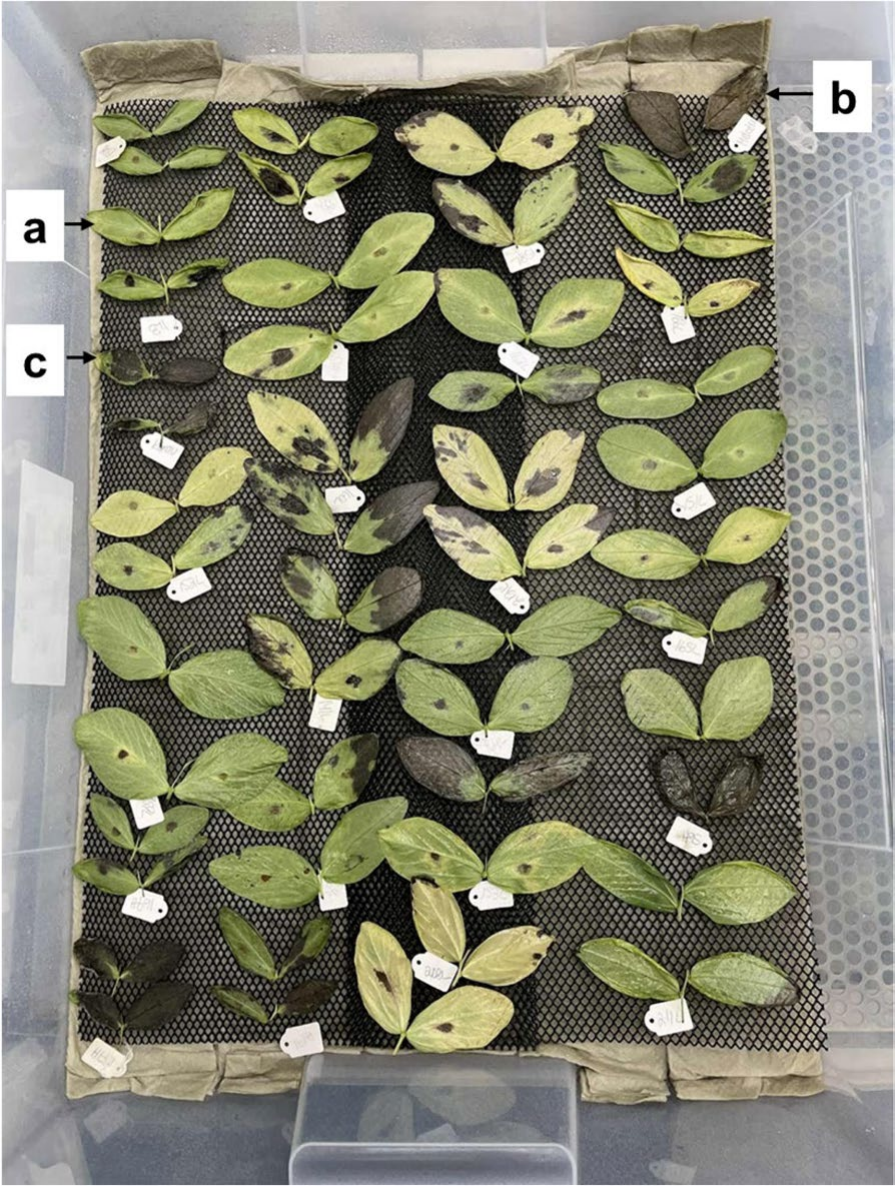
Setup of the detached leaf assay for *Botrytis* infection screening, indicating the partially resistant genotype ILB 938/2 (**a**), susceptible genotype Mélodie/2 (**b**); highly susceptible cv. Kontu (**c**); and some of the RIL population

For disease screening, we adapted our recently developed detached leaflet assay [13]. Fully expanded leaves, each with four leaflets, were collected from nodes four to eight of seven- to eight-week-old plants of the RILs, two hours prior to inoculation, and kept in sterile moist tissue. Five layers of sterile paper towels were arranged in a plastic box (78 cm × 56 cm × 18 cm, 55 L), one liter of sterile water was poured on the paper towels, and a plastic net was placed so the leaf blades did not rest on the wet surface (Fig. 1). The leaves were arranged on the nets in a randomized complete block design (RCBD) with four replicates, with the plastic box as a replicate. Leaves of ILB 938/2, Mélodie/2, and Kontu were included as controls in each box. A 25 μl aliquot of the spore suspension of each of the four *Botrytis* species was inoculated using a pipette onto the leaflets in a clockwise manner, so that each leaflet received inoculum nearly simultaneously. The boxes were placed in the growth chamber and incubated for seven days at 22 °C and 12 h photoperiod with 170–188 μmol m⁻^2^ s⁻^1^ PPFD. To maintain the humidity, sterile water was sprayed 2–3 times every day inside the boxes. Visible symptoms of the fungal infection and necrosis were monitored every day. After 7 days, the symptoms on leaflets were scored following a 1–9 disease scale, where, 1: no necrotic lesion and/or chlorosis (complete resistance); 2: 1.0–12.5% necrotic lesion and/or chlorosis (high resistance); 3: 13.0–25.5% necrotic lesion and/or chlorosis (moderate resistance); 4: 26.0–38.5% necrotic lesion and/or chlorosis (low resistance); 5: 39.0–51.5% necrotic lesion and/or chlorosis (low susceptibility); 6: 52.0–64.5% necrotic lesion and/or chlorosis (moderate susceptibility); 7: 65.0–77.5% necrotic lesion and/or chlorosis (high susceptibility); 8: 78.0–90.5% necrotic lesion and/or chlorosis (very high susceptibility) and 9: > 91.0% (severe susceptibility) [28].

### Map construction and QTL mapping

The mapping population was genotyped using the Axiom “Vfaba_v2” 60K array [22, 29]. The genetic map was originally produced by Gela et al. [17]. The linkage map was constructed with MapDisto v. 1.7.7.0.1 [30], applying a logarithm of odds (LOD) score of 3.0 and a recombination fraction of 0.35. The Kosambi function was used to calculate the map distance in centimorgans (cM) [31]. A final genetic map was constructed from 4,089 SNP markers, which mapped to six linkage groups (LGs) representing the six chromosomes of faba bean [17]. The LGs were assigned to faba bean chromosomes according to the faba bean physical map [1]. The orientation of SNP markers on the original LG1 [17] was reversed to match the orientation of the faba bean physical map, while the remaining linkage groups required no such change.

QTL mapping was performed using composite interval mapping (CIM) implemented in R/qtl v.1.50 [32]. QTL LOD significance thresholds were determined by permutation testing (1,000 permutations) using the scanone function at a significance level of *P* = 0.05, following standard procedures. The percentage of the phenotypic variance explained, and additive effects of detected QTLs were estimated using the fitqtl function. SNP marker positions were visualized by MapChart v. 2.2 [33]. The confidence intervals for each QTL were calculated using the 1.5-LOD support interval implemented in the lodint function. To identify candidate genes, the coding sequences of the SNP markers in the QTL intervals were searched by BLASTn in Phytozome v14 against the Vicia faba v1.1 assembly (https://phytozome-next.jgi.doe.gov/info/Vfaba_v1_1).

### Statistical analysis

The JMP Pro 14 statistical computing program was used to analyze the phenotyping data set [34]. Data was subjected to two-way analysis of variance (ANOVA) to determine the effects of RILs, *Botrytis* species, and their interactions. Principal component analysis (PCA) was performed using RIL means with R version 4.5.1 [35] with package ggplot2 [36].

## Results

### Phenotypic variation

**Fig. 2:**
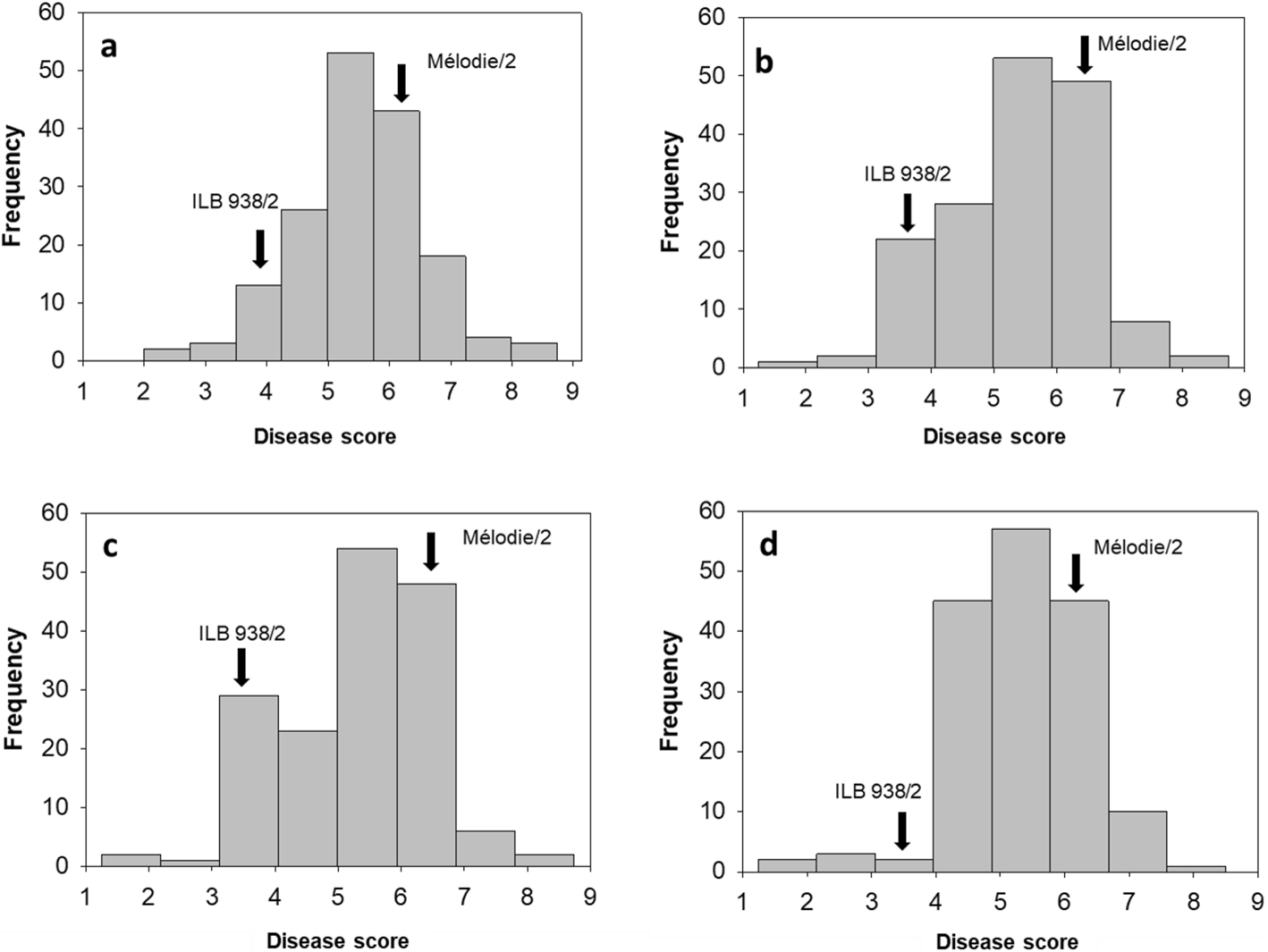
Frequency distributions of the disease scores when four *Botrytis* isolates were inoculated onto 165 RILs derived from the cross Mélodie/2 × ILB 938/2 at the F8 generation. **a** *B. fabae* (19B053-4); **b** *B. cinerea* (19B048); **c** *B. pseudocinerea* (18B11); and **d** *B. fabiopsis* (19B175)

**Fig. 3:**
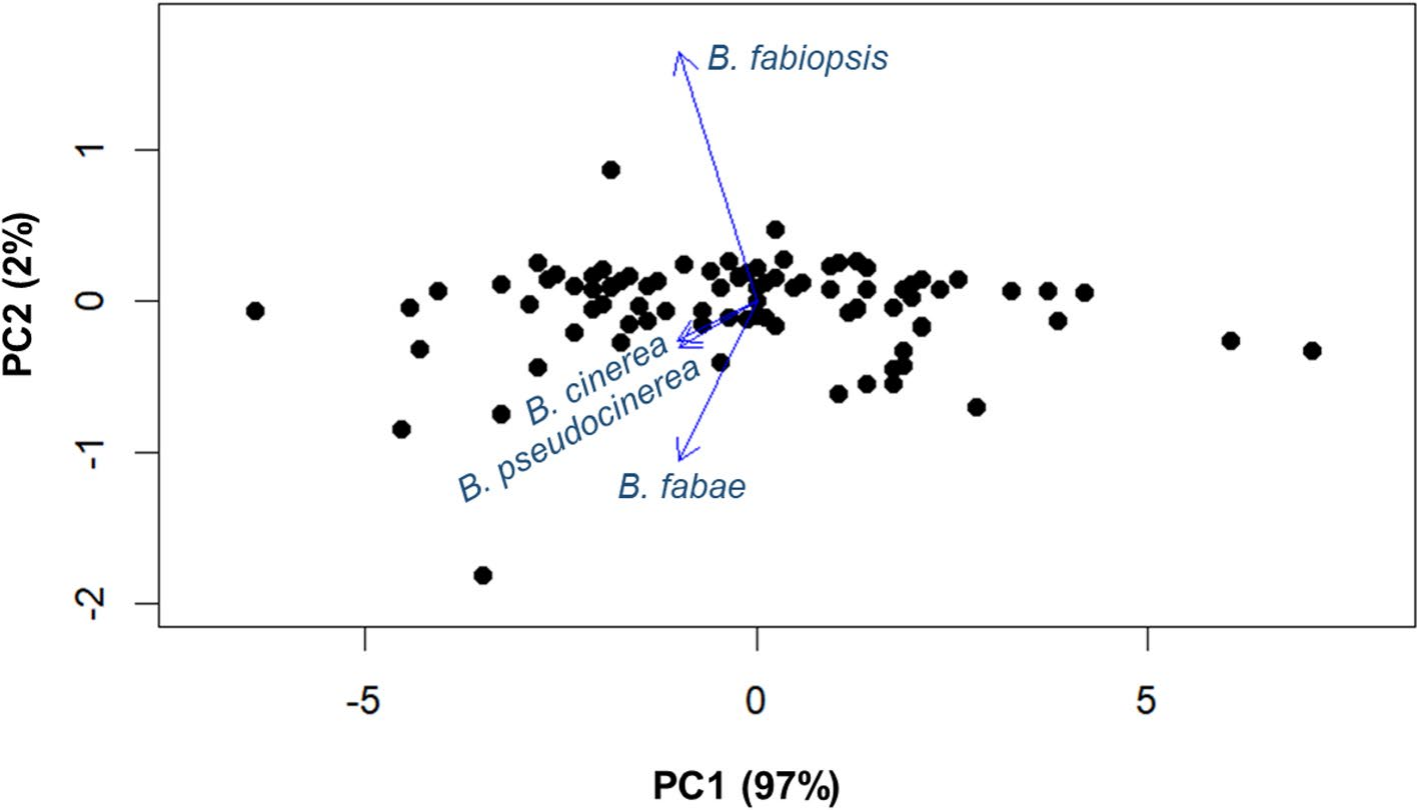
PCA-biplot graph of the first two principal components (PC1 vs PC2) for four *Botrytis* species across 165 RILs

RILs showed significant variation in CS disease severity score caused by the isolates of *B. fabae*, *B. cinerea*, *B. pseudocinerea*, and *B. fabiopsis* (*P* < 0.0001). The interaction between RILs and *Botrytis* species was also significant (Table 1). The parental lines of the RIL population (Mélodie/2 and ILB 938/2) had significant differences in disease severity score to CS across all four *Botrytis* species (Fig. 2). Mélodie/2 showed moderate susceptibility to CS caused by four *Botrytis* species, whereas ILB 938/2 exhibited partial resistance to CS across the *Botrytis* species. The pattern of disease severity in response to CS evaluation for the RILs displayed a continuous variation across different *Botrytis* isolates (Fig. 2), suggesting that the polygenic nature of CS severity and CS resistance is quantitatively inherited. The PCA plot shows the distribution and correlation among the scores of the four isolates on the 165 RILs. The first two principal components explained 99% of the total variation. The high proportion of variance explained by the first principal component is expected and reflects the strong correlations among four isolates (Fig. 3).

**Table 1:** Analysis of variance of disease severity scores (1–9 scale) of 165 RILs using four *Botrytis* species

Source	df	Mean square	*P-*value
Block	3	5.176	<0.0001
RILs	164	17.853	<0.0001
*Botrytis* species	3	6.343	<0.0001
RILs x *Botrytis* species	492	0.204	<0.0001
Error	1977	0.127	

*df* Degrees of freedom, *RILs* Recombinant inbred lines

### QTL analysis

**Fig. 4:**
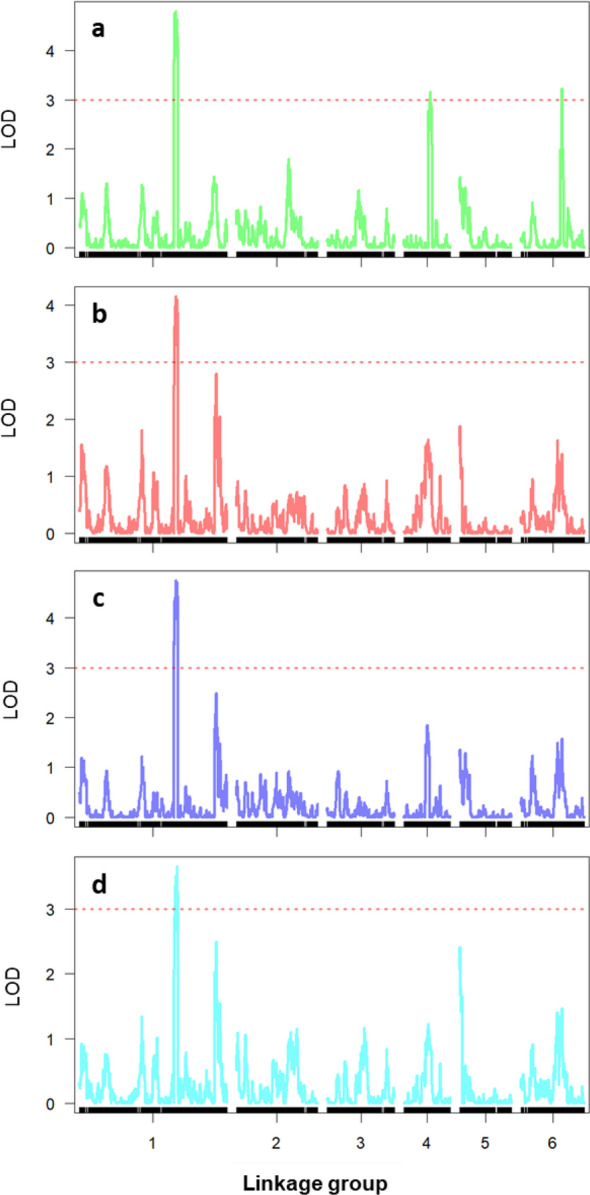
Genomic regions associated with *Botrytis* resistance in faba bean showing the QTL peak locations. **a** *B. fabae* (19B053-4); **b** *B. cinerea (*19B048*)*; **c** *B. pseudocinerea* (18B11); and **d** *B. fabiopsis* (19B175)

Six QTLs associated with the infection responses to the four *Botrytis* isolates were detected across Chr1, Chr4, and Chr6 (LG1, LG4, and LG6). The strongest signals for all four *Botrytis* species were located on LG1, where q-BF1 (*B. fabae*), q-BC1 (*B. cinerea*), q-BPC1 (*B. pseudocinerea*), and q-BFsis1 (*B. fabiopsis*) all mapped to 254–265 cM (580–663 Mb of faba bean Chr1L), with LOD scores ranging from 3.66 to 4.79 and R^2^ values between 8.5% and 10.8%. Two additional QTLs for *B. fabae* response were identified on LG4 (q-BF4, 825–918 Mb of faba bean Chr4) and LG6 (q-BF6, 997–1025 Mb of faba bean Chr6) (Table 2; Fig. 4). The physical marker positions are presented in Table S1. Positive additive values for all QTLs suggest that Mélodie/2 was the donor of susceptibility alleles (Table 2).

**Table 2:** Quantitative trait loci (QTLs) for four *Botrytis* isolates from the cross Mélodie/2 × ILB 938/2

QTL	LG	QTL Peak (cM)	1.5-LOD interval (cM)	LOD score	R^2^	Add
q-BF1	1	259.2	254.89—263.51	4.79	10.8	0.36
q-BF4	4	70.6	65.71—75.00	3.15	7.1	0.23
q-BF6	6	110.4	107.63—111.96	3.22	7.2	0.25
q-BC1	1	259.0	255.19—264.78	4.15	10.1	0.34
q-BPC1	1	259.0	255.81—263.51	4.75	10.7	0.35
q-BFsis1	1	263.0	255.19—264.78	3.66	8.6	0.31

QTL abbreviations: q-BF, *B. fabae* (19B053-4); q-BC, *B. cinerea* (19B048); q-BPC, *B. pseudocinerea* (18B11); q-BFsis: *B. fabiopsis* (19B175). LG: Linkage Group, R^2^, Percentage of phenotypic variance explained by QTL; Add, Additive genetic effect

### Identification of putative candidate genes

**Fig. 5:**
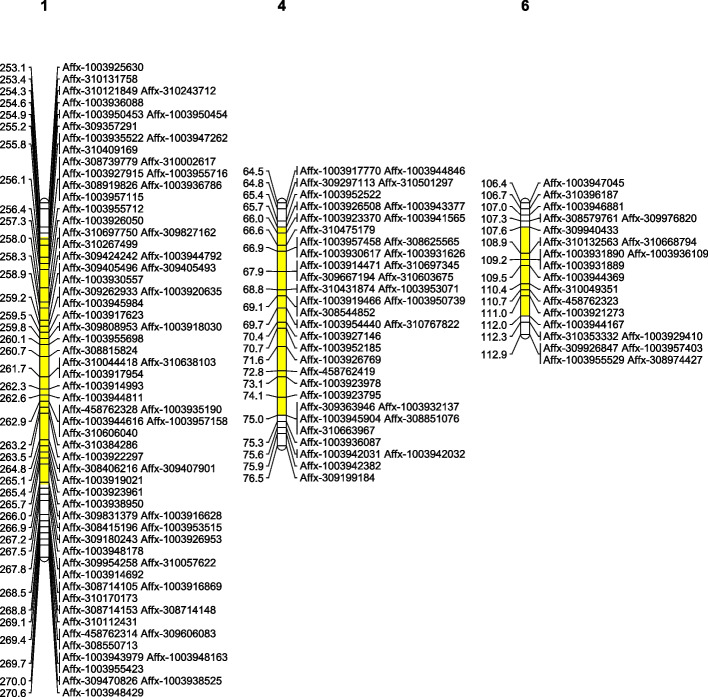
Location of *Botrytis* resistance genomics regions on segments of faba bean chromosomes 1L (*q-BF1*, *q-BC1*, *q-BPC1*, and *q-BFsis1*), 4 (*q-BF4*), and 6 (*q-BF6*)

All three QTL intervals on LG1, LG4, and LG6 (Fig. 5) included clusters of putative candidate genes related to plant disease and defence-related (Table S1). In LG1, the regions covered by overlapping QTLs q-BF1, q-BC1, q-BPC1, and q-BFsis1 included members of gene families for F-box proteins, DnaJ/Hsp40 chaperones, serine proteases, S-adenosylmethionine synthetases, chloride channels, SBP/MYB/FAR1 transcription factors, nodulin-like MFS transporters, lipases, and leucine-rich pentatricopeptides. Within the LG4 q-BF4 region were found genes annotated as a WRKY transcription factor, zeaxanthin epoxidase, Caffeoyl-CoA O-methyltransferase, subtilisin-like protease, RING/U-box protein, casein kinase II α1, pectin methylesterase, aspartyl protease, peroxidase, and scarecrow-like protein. LG6 q-BF6 contained members of five classical and strongly defense-related gene families, namely AP2/ERF, LRR, RING/U-box E3 ligases, thioredoxin, and hydroxyproline-rich glycoproteins.

## Discussion

This is the first study to genetically map the responses of faba bean to four *Botrytis* species. We found a single region on faba bean Chr1 hosting loci for resistance to *B. fabae*, *B. cinerea*, *B. pseudocinerea*, and *B. fabiopsis*. *B. fabae* response had additional QTLs on Chr4 and Chr6. Notably, CS resistance QTLs on Chr1 and Chr6 were also reported by Gela et al. [17] using the same mapping populations and marker system but different screening methods and a different isolate of *B. fabae*. The lack of species‑specific QTLs for the non‑*B.* *fabae* isolates may reflect either a genuinely shared host‑response mechanism or limitations in either isolate virulence or mapping power. These results suggest a potentially shared genetic basis of resistance across *Botrytis* species, but this requires validation under field conditions and in additional genetic backgrounds. Putative disease- and defense-related candidate genes were identified within our identified QTL regions using faba bean genome gene annotations, some playing critical roles in resistance to *Botrytis* species. However, their involvement in CS resistance in faba bean remains hypothetical and will require functional validation.

In our study, using the detached leaf assay, we successfully confirmed genomic regions previously detected through whole-plant screening under different indoor growing conditions in Saskatchewan for *B. fabae* [17]. The phenotypic data also showed a moderate positive correlation between RILs screened using the whole-plant and the detached leaf assays (R^2^ = 0.480; Figure S1). It should be noted that the detached leaf (this study) and whole-plant assays (Gela et al. [17]) used different *B. fabae* isolates and experimental conditions, and are therefore not directly comparable. This indicates that the detached leaf assay is an efficient alternative to whole-plant screening for *Botrytis* resistance under indoor climatic control conditions in this species, particularly for genetic mapping studies. It is rapid, space-efficient, and allows controlled infection with precise scoring of lesion development on excised leaves [37]. Field screening, although challenging, remains essential for evaluating the overall plant response, including plant–pathogen interactions and genotype-by-environment effects [38]. The detached leaf assay provides a valuable complementary approach to whole-plant screening.

In this study, six QTLs associated with *Botrytis* infection were identified, five of which correspond to previously reported loci, underscoring the consistency of these genomic regions in contributing to CS resistance in faba bean. QTLs q-BF1, q-BC1, q-BPC1, and q-BFsis1 were mapped to the same region of Chr1, in the region for q-BF1.2 reported by Gela et al. [17]. Our q-FB6 was found to be identical to QTL q-FB6.1 reported by Gela et al. [17]. These overlaps confirm the stability of these loci across different growing conditions and *Botrytis* isolates. Similarly, Webb et al. [18] identified a large interval for *B. fabae* resistance on Chr1, consistent with our findings, though their additional QTLs on Chr3 and Chr5 may reflect both environmental variation and the use of a different genetic background (cv. Maris Bead). Skovbjerg et al. [25] reported marker-trait associations for *B. fabae* response located at the beginning of Chr1, whereas our significant peaks were near the end of this chromosome. Notably, we also detected a novel QTL on Chr4 for *B. fabae*, which has not been reported before. The detected minor-effect QTLs confirm the partial resistance to *Botrytis* infection derived from ILB 938/2, Icarus, and cv. Maris Bead, which has been used in genetic mapping efforts. Beyond these sources, additional resistance germplasm has been identified [19, 22]. Further efforts are needed to study the genetic architecture and loci of these sources in order to develop the means for durable gene pyramiding and the breeding of broad-spectrum *Botrytis* resistance in this crop.

The QTLs and associated SNP markers identified in this study are specific to the segregating alleles present in the Mélodie/2 × ILB 938/2 mapping population. As with most bi-parental QTL studies, the detected loci reflect resistance mechanisms contributed by these two parental genotypes and may not be directly transferable to other genetic backgrounds without validation. Previous studies in faba bean have similarly reported population-specific QTLs for chocolate spot resistance, with partial overlap among mapping populations but substantial differences in the genomic regions detected [17, 18, 25]. The relatively modest proportion of phenotypic variance explained by individual QTLs in this study (7–11%) is consistent with the quantitative and polygenic nature of *Botrytis* resistance in faba bean, where resistance is controlled by multiple minor-effect loci rather than single major genes. Consequently, while the identified loci provide valuable targets for marker development and functional validation, their direct application in marker-assisted selection may be limited. Instead, these results support the use of genomic selection or QTL-informed genomic prediction approaches, which are better suited to capturing the cumulative effects of numerous small-effect loci underlying durable disease resistance.

The annotated faba bean genome facilitated the identification of candidate genes associated with the QTLs that were identified. Several of the candidate gene families identified in this study overlap with those reported by Webb et al. [18]. Both studies found, within the mapping intervals, genes for F-box and U-box–type E3 ubiquitin ligases, thioredoxin, and chaperone (DnaJ/Hsp40) proteins, which potentially involved in protein turnover and stress signalling during pathogen attack. Similarly, transcription factor families including AP2/ERF, MYB, and SBP were identified as putative candidate genes, given their known regulatory roles in activating defence-related genes. Moreover, genes for peroxidases, lipases, and S-adenosylmethionine synthetase identified in the genome assembly for our QTL regions may correspond to oxidative stress and secondary metabolism genes identified in Webb et al. [18]. Consistent with this, Castillejo et al. [39] reported that resistance to *B. fabae* in faba bean is associated with a more efficient Photosystem II repair cycle and enhanced redox regulation, further highlighting the likely importance of oxidative balance in *Botrytis* resistance. Direct evidence linking specific candidate genes to *Botrytis* resistance in legumes remains limited. However, studies in chickpea (*Cicer arietinum* L.) have reported the involvement of peroxidases, WRKY transcription factors, and antioxidant enzymes in defense responses to *B. cinerea* infection [40–42]. These genes are known to mediate oxidative stress regulation and pathogen-induced signaling pathways in legumes [43]. In Arabidopsis, functional studies have demonstrated that certain F-box proteins and WRKYs play critical roles in *B. cinerea* resistance by modulating jasmonate and salicylic acid pathways [44, 45], supporting the possibility of conserved defense role of these gene families across species. Accordingly, candidate genes within the detected QTL intervals, particularly those with annotated roles in plant defense or stress responses, represent putative targets for functional validation and may underlie *Botrytis* resistance.

## Conclusions

We confirmed previously reported minor-effect QTLs and identified a novel QTL associated with *Botrytis* resistance in faba bean. These loci provide valuable targets for marker-assisted and genomic selection as well as for functional verification. The detached leaf assay proved to be an efficient method for disease screening in genetic mapping studies. The identified candidate genes may support further dissection of the biological mechanisms underlying *Botrytis* resistance. Future work on mapping additional resistance sources and combining QTLs to enhance durable resistance in breeding programs would also be highly worthwhile.

## Supplementary Information

Supplementary Material 1.

Supplementary Material 2.

## Data Availability

The datasets used and/or analyzed during the current study are available from the corresponding author on reasonable request.
