# Supplementary material for: QTL analysis uncovers the genetic architecture of resistance to chocolate spot disease caused by four Botrytis species on faba bean
Source: BMC Plant Biol. 2026 Apr 10;26:848. doi: 10.1186/s12870-026-08699-0 (PMC13173894; doi:10.1186/s12870-026-08699-0)
Supplement: Supplementary file 1 — Supplementary Material 1. [file 12870_2026_8699_MOESM1_ESM.pptx]

## Slide 1
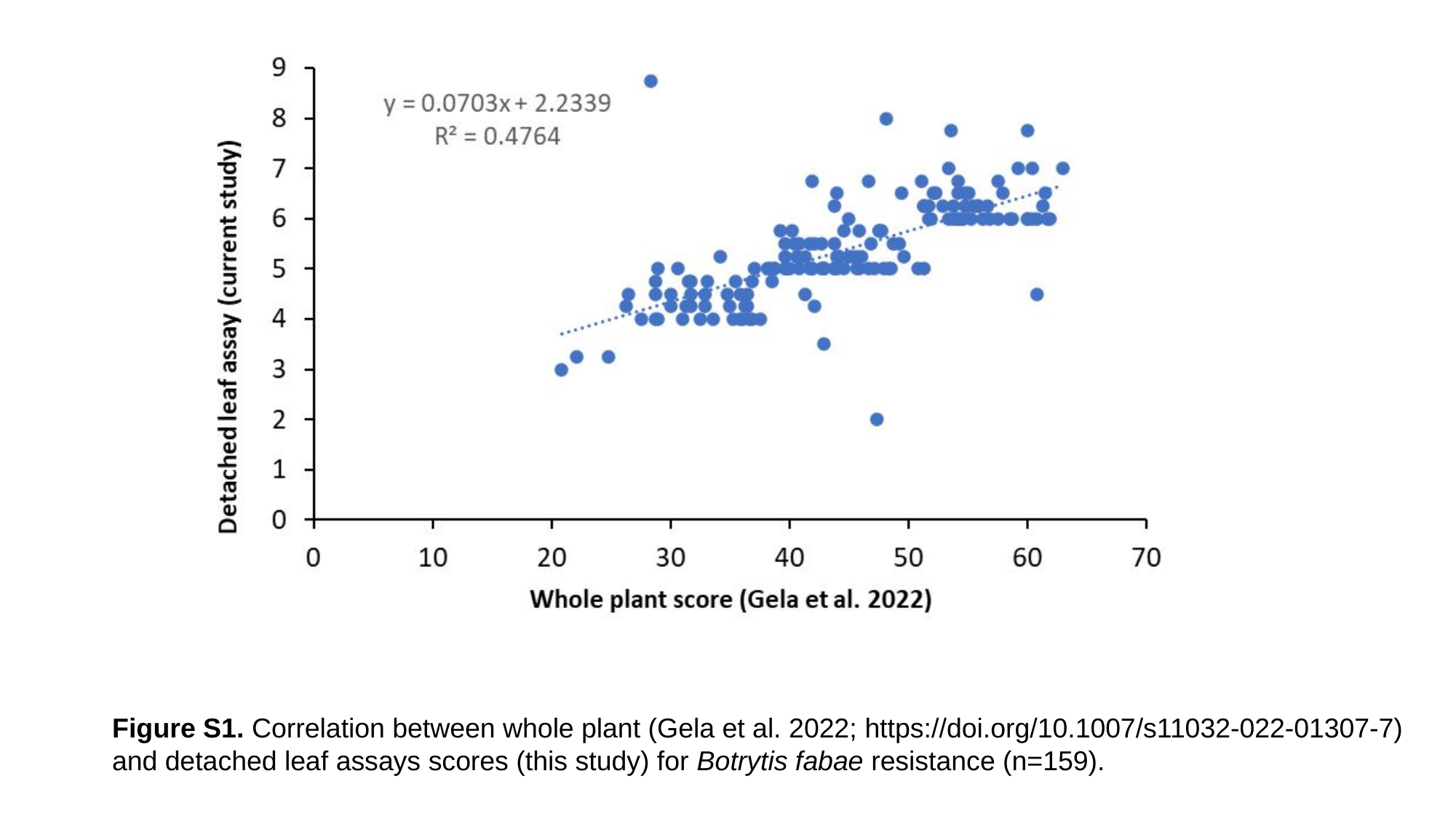

Figure S1. Correlation between whole plant (Gela et al. 2022; https://doi.org/10.1007/s11032-022-01307-7) and detached leaf assays scores (this study) for Botrytis fabae resistance (n=159).
